# Association of Caffeine Consumption and Brain Amyloid Positivity in Cognitively Normal Older Adults

**DOI:** 10.3233/JAD-220591

**Published:** 2023-05-16

**Authors:** Yong-Bo Zheng, Jie Sun, Le Shi, Si-Zhen Su, Xuan Chen, Qian-Wen Wang, Yue-Tong Huang, Yi-Jie Wang, Xi-Mei Zhu, Jian-Yu Que, Na Zeng, Xiao Lin, Kai Yuan, Wei Yan, Jia-Hui Deng, Jie Shi, Yan-Ping Bao, Lin Lu

**Affiliations:** a Peking University Sixth Hospital, Peking University Institute of Mental Health, NHC Key Laboratory of Mental Health (Peking University), National Clinical Research Center for Mental Disorders (Peking University Sixth Hospital), Chinese Academy of Medical Sciences Research Unit (No. 2018RU006), Peking University, Beijing, China; b Peking-Tsinghua Center for Life Sciences and PKU-IDG/McGovern Institute for Brain Research, Peking University, Beijing, China; c Pain Medicine Center, Peking University Third Hospital, Beijing, China; d The First Affiliated Hospital of Xinxiang Medical University, Henan, China; e National Institute on Drug Dependence and Beijing Key Laboratory of Drug Dependence, Peking University, Beijing, China; f School of Public Health, Peking University, Beijing, China

**Keywords:** Amyloid positivity, caffeine, dementia, sex-specific

## Abstract

**Background:**

Several epidemiological studies have reported the protective role of caffeine on health outcomes; however, it remained debatable on caffeine consumption and brain amyloid positivity.

**Objective:**

We aimed to determine the relationship between caffeine consumption and brain amyloid pathology in cognitively normal older adults.

**Methods:**

The dataset used for analysis in this cross-sectional study was selected from the Anti-Amyloid Treatment in Asymptomatic Alzheimer’s (A4) Study. Multivariable logistic regression analyses were performed to explore the association between caffeine consumption and amyloid positivity using odds ratios (ORs) and 95% confidence intervals (CIs).

**Results:**

In total, 4,394 participants were included in the final analysis. No significant association between caffeine consumption and amyloid positivity was observed in the whole participants (OR, 0.95; 95% CI, 0.78–1.14; *p* = 0.558). Subgroup analysis showed that caffeine intake was significantly associated with decreased amyloid positivity in males (OR, 0.72; 95% CI, 0.54–0.97; *p* = 0.032) but not in females (OR, 1.14; 95% CI, 0.90–1.46; *p* = 0.280), and the association between caffeine and amyloid positivity was not affected by age or *APOE* genotypes. In addition, different levels of caffeine were not associated with amyloid positivity.

**Conclusion:**

The findings suggest that caffeine consumption was not significantly associated with amyloid positivity in the whole sample. However, caffeine consumption may be inversely associated with amyloid positivity among males but not females. More studies are needed to explore the mechanisms underlying caffeine consumption and brain amyloid positivity.

## INTRODUCTION

Caffeine, a central nervous system stimulant of the methylxanthine class, occurs naturally in coffee, tea, and cocoa [1]. It has been consumed for hundreds of years and has become an important part of cultural traditions and social life [2]. It is the most extensively consumed psychoactive substance [3] and is legal and unregulated in almost all parts of the world. It is estimated that adults consume an average of nearly two cups of coffee daily, which is the most typical beverage containing caffeine [4]. Caffeine consumption can affect multiple health outcomes [5], and has beneficial effects on various systems, including cardiology [6], endocrinology [7], psychiatry [8], and neurology [9].

Epidemiological studies have reported protective effects of caffeine against Alzheimer’s disease (AD) [10–12] and cognitive decline [13, 14]. However, there is limited information on the neuropathological evidence supporting the protective effects of caffeine against AD and related cognitive decline in humans. Preclinical findings indicate that caffeine utilization can reduce amyloid-β (Aβ) levels in mammalian animals, particularly in male rats [15–17]. A study indicated that lifetime coffee intake of ≥2 cups/day was significantly associated with a lower Aβ positivity in the human brain, compared to coffee intake of <2 cups/day [18]. Another cohort study also found that higher coffee consumption is associated with slower cognitive decline and less cerebral Aβ accumulation over 126 months [19].

As a biomarker for dementia, detecting Aβ in cognitively normal older adults has clinical implications for the prevention and intervention of cognitive decline and dementia in their early stage [20–22]. Although great progress has been made in exploring the mechanisms underlying the relationship between caffeine and amyloid positivity, the relationship between caffeine intake and amyloid pathology in the human brain in the stratified populations remains unclear. Accordingly, we aimed to determine the relationship between caffeine intake and brain amyloid pathology in cognitively normal older adults. Moreover, we aimed to determine the magnitude of the effect of caffeine intake on amyloid positivity among population-stratified participants.

## METHODS

### Participants

The dataset used for analysis in this cross-sectional study was selected from the Anti-Amyloid Treatment in Asymptomatic Alzheimer’s (A4)/Longitudinal Evaluation of Amyloid Risk and Neurodegeneration (LEARN) Study, which was conducted at 67 clinical trial sites in the US, Canada, and Japan [23]. The A4 study is a secondary prevention trial of an anti-amyloid antibody in clinically normal individuals aged 65–85 years with elevated brain Aβ peptides on positron emission tomography (PET), as described elsewhere [24]. The inclusion criteria comprised complete information on caffeine consumption, demographic information, habits, apolipoprotein E (*APOE*) genotypes and comorbidities, a score between 25 and 30 on the Mini-Mental State Examination [25], and a global Clinical Dementia Rating (CDR) scale score of 0 [26]. This study was approved by the institutional review boards of all participating institutions, and written informed consent was obtained from all participants. This study followed the Strengthening the Reporting of Observational Studies in Epidemiology (STROBE) reporting guidelines. To apply for the use of A4 data, the study was also approved by the Institutional Review Board of the Peking University Sixth Hospital.

### Assessment of caffeine intake

Participants were asked to report their daily consumption of caffeine-containing beverages using the average number of cups of caffeine consumed daily. Participants were considered as caffeine drinkers (≥1 cup/day) and non-caffeine drinkers (0 cups/day) based on their daily consumption of caffeine-containing beverages.

### Amyloid PET neuroimaging

Amyloid PET neuroimaging was performed at baseline in the A4/LEARN study. Cerebral Aβ levels were determined using post-processed Aβ tracer florbetapir (^18^F-AV-45) PET imaging data. PET data processing was conducted by Invacare LLC. We used the continuous composite total cerebral amyloid tracer standardized uptake value ratio (SUVR), calculated using the whole cerebellum as the reference region. Aβ positivity was defined as an ^18^F-florbetapir PET SUVR ≥1.10, while Aβ negativity was defined as an SUVR <1.10, as previously described [27, 28].

### Covariates

Covariates contained demographic information, including age, sex, body mass index (BMI), marital status, and educational attainment; habits, including alcohol consumption and smoking; *APOE* genotypes; and comorbidities, including psychiatric, neurological, cardiovascular, and endocrinological diseases. A summary of covariates is provided in Supplementary Table 1. According to previous literature [29], age was categorized into 5-year age groups as follows: 65–70, 70–75, 75–80, and 80–85 years. Based on the criteria established by the National Institute of Health [30], the BMI cut-off values for underweight, normal weight, overweight, and obesity were <18.5, 18.5–24.9, 25.0–29.9, and ≥30 kg/m^2^, respectively. The *APOE* genotypes were ɛ2/ɛ2, ɛ2/ɛ3, ɛ2/ɛ4, ɛ3/ɛ3, ɛ3/ɛ4, and ɛ4/ɛ4. The most common *APOE* allele, *APOE* ɛ3, was associated with an average risk of AD, whereas the *APOE* ɛ4 and *APOE* ɛ2 alleles lead to higher and lower AD risks, respectively [31, 32].

### Statistical analysis

Descriptive statistics were used to present the demographic and clinical characteristics of participants according to status of caffeine drinking. Independent sample *t*-tests and χ^2^ tests were used to compare continuous and categorical variables, respectively.

Multivariable logistic regression analyses were performed to explore the association between caffeine consumption and amyloid positivity. Model 1 included sex, age, and *APOE* genotypes as covariates, whereas model 2 included demographic information, habits, *APOE* genotypes, and comorbidities as covariates. To explore the sex, age, and *APOE* genotypes effect of caffeine consumption on amyloid positivity, interactions included sex×caffeine, age×caffeine, *APOE* genotypes×caffeine, were further added to the multivariable logistic regression analysis (model 3). When the interaction was significant, a multivariable logistic regression analysis was further conducted to assess the caffeine consumption and amyloid positivity in subgroup participants. Similarly, we performed the multivariable logistic regression models mentioned above to explore the association between cups of caffeine consumption and amyloid positivity in all participants. Odds ratios (ORs) and 95% confidence intervals (CIs) were calculated for all regression analysis. Two-sided Wald tests were performed to determine whether the ORs in the regression models were significant. A *p*-value <0.05 was considered statistically significant. All statistical analyses were performed using SPSS statistical software version 22 (IBM Corp).

## RESULTS

### Demographic and clinical characteristics of participants

**Fig. 1:**
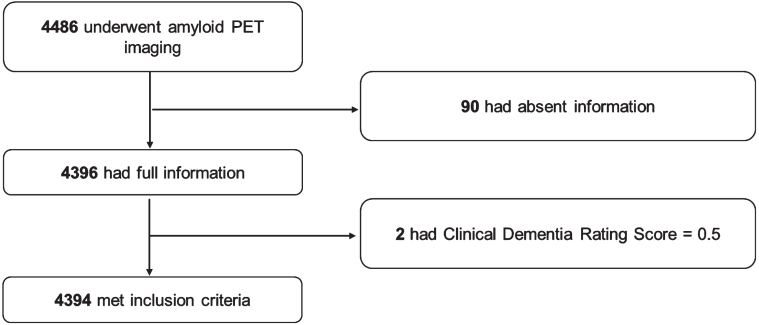
Flow chart of the process of sample inclusion.

A total of 4,486 participants underwent PET. Of this sample, 4,396 had available information on caffeine consumption, demographic information, habits, *APOE* genotypes, and comorbidities. Two participants were excluded for preclinical dementia with a CDR of 0.5, and 4,394 participants were included in the present analysis. The detailed process of sample inclusion is shown in Fig. 1.

Among the included participants, 3,696 were caffeine drinkers, and 698 were non-caffeine drinkers. The mean age of included participants was 71.29±4.67 years. The following factors were found to be significantly different between caffeine-drinkers and non-caffeine drinkers: racial category, and alcohol consumption per day. The detailed demographic and clinical characteristics of participants according to caffeine-drinkers are shown in Table 1.

**Table 1:** Demographic and clinical characteristics of participants

	Total	Non-caffeine drinkers	Caffeine drinkers	*p*
Number of observations (N [% ])	4,394 (100.0)	698 (100.0)	3,696 (100.0)
Sex (N [% ])				0.390
Male	17,83 (40.6)	273 (39.1)	15,10 (40.9)
Female	2,611 (59.4)	425 (60.9)	2,186 (59.1)
Age (mean [SD])	71.29±4.67	71.42±4.62	71.26±4.68	0.425
Age (N [% ])				0.932
65–70 y	2,087 (47.5)	331 (47.4)	1,756 (47.5)
70–75 y	1,407 (32)	222 (31.8)	1,185 (32.1)
75–80 y	640 (14.6)	100 (14.3)	540 (14.6)
80–85 y	260 (5.9)	45 (6.4)	215 (5.8)
BMI (mean [SD])	27.50±5.10	27.45±5.44	27.51±5.04	0.779
BMI (N [% ])				0.352
Underweight (<18.5 kg/m^2^)	32 (0.7)	7 (1.0)	25 (0.7)
Normal weight (18.5–24.9 kg/m^2^)	1,469 (33.4)	249 (35.7)	1,220 (33.0)
Overweight (25.0–29.9 kg/m^2^)	1,766 (40.2)	264 (37.8)	1,502 (40.6)
Obesity (≥30.0 kg/m^2^)	1,127 (25.6)	178 (25.5)	949 (25.7)
Marital status (N [% ])				0.614
Married	3,107 (70.7)	488 (69.9)	2,619 (70.9)
Not married	1,287 (29.3)	210 (30.1)	1,077 (29.1)
Education (y, mean [SD])	16.58±2.84	16.55±2.64	16.59±2.87	0.736
Racial categories (N [% ])				<0.001
American Indian or Alaskan Native	9 (0.2)	0 (0)	9 (0.2)
Asian	169 (3.8)	22 (3.2)	147 (4.0)
Native Hawaiian or other Pacific Islander	2 (<0.1)	0 (0)	2 (0.1)
Black or African American	155 (3.5)	47 (6.7)	108 (2.9)
White	4,008 (91.2)	621 (89.0)	3,387 (91.6)
Unknown or not reported	51 (1.2)	8 (1.1)	43 (1.2)
Alcohol drinking per day (N [% ])				<0.001
None	2,222 (50.6)	484 (69.3)	1,738 (47.0)
At least one cup	2,172 (49.4)	214 (30.7)	1,958 (53.0)
Smoking per day (N [% ])				0.086
None	4,340 (98.8)	694 (99.4)	3,646 (98.6)
At least one package	54 (1.2)	4 (0.6)	50 (1.4)
*APOE* genotype (N [% ])				0.436
ɛ2/ɛ2	24 (0.5)	6 (0.9)	18 (0.5)
ɛ2/ɛ3	446 (10.2)	65 (9.3)	381 (10.3)
ɛ2/ɛ4	114 (2.6)	12 (1.7)	102 (2.8)
ɛ3/ɛ3	2,393 (54.5)	385 (55.2)	2,008 (54.3)
ɛ3/ɛ4	1,279 (29.1)	209 (29.9)	1,070 (29.0)
ɛ4/ɛ4	138 (3.1)	21 (3.0)	117 (3.2)
Psychiatric disorders (N [% ])				0.058
No	3,332 (75.8)	549 (78.7)	2,783 (75.3)
Yes	1,062 (24.2)	149 (21.3)	913 (24.7)
Neurological diseases (N [% ])				0.095
No	3,393 (77.2)	522 (74.8)	2,871 (77.7)
Yes	1,001 (22.8)	176 (25.2)	825 (22.3)
Cardiovascular diseases (N [% ])				0.180
No	1,705 (38.8)	255 (36.5)	1,450 (39.2)
Yes	2,689 (61.2)	443 (63.5)	2,246 (60.8)
Endocrinological diseases (N [% ])				0.183
No	2,311 (52.6)	351 (50.3)	1,960 (53.0)
Yes	2,083 (47.4)	347 (49.7)	1,736 (47.0)
Amyloid (N [% ])				0.599
Not elevated	2,896 (65.9)	454 (65.0)	2,442 (66.1)
Elevated	1,498 (34.1)	244 (35.0)	1,254 (33.9)

*APOE*, apolipoprotein E; BMI, body mass index; and NA, not available.

### Relationship between caffeine consumption and amyloid positivity

Table 2 presents the results of relationship between caffeine consumption and amyloid positivity in all participants. The proportions of amyloid positivity in caffeine and non-caffeine drinkers were 33.9% and 35.0%, respectively, in all participants. No significant association was found between caffeine consumption and amyloid positivity (model 1: OR, 0.96; 95% CI, 0.80–1.15; *p* = 0.627; and model 2: OR, 0.95; 95% CI, 0.79–1.14; *p* = 0.558).

**Table 2:** Relationship between caffeine consumption and amyloid positivity in all participants

Factors	Model 1 adjusted OR (95% CI)	*p*	Model 2 adjusted OR (95% CI)	*p*	Model 3 adjusted OR (95% CI)	*p*
Caffeine consumption (ref: no)	0.96 (0.80, 1.15)	0.627	0.95 (0.78, 1.14)	0.558	0.67 (0.05, 8.30)	0.752
Female (ref: male)	1.09 (0.95, 1.25)	0.217	1.09 (0.93, 1.26)	0.286	0.78 (0.55, 1.10)	0.160
70–75 y (ref: 65–70 y)	**1.54 (1.31, 1.80)**	**<0.001**	**1.54 (1.31, 1.80)**	**<0.001**	**1.56 (1.06, 2.29)**	**0.026**
75–80 y (ref: 65–70 y)	**2.30 (1.89, 2.81)**	**<0.001**	**2.31 (1.88, 2.83)**	**<0.001**	**2.06 (1.24, 3.40)**	**0.005**
80–85 y (ref: 65–70 y)	**2.70 (2.04, 3.58)**	**<0.001**	**2.72 (2.05, 3.63)**	**<0.001**	**3.33 (1.71, 6.50)**	**<0.001**
ɛ2/ɛ3 (ref: ɛ2/ɛ2)	1.06 (0.35, 3.21)	0.921	1.07 (0.35, 3.26)	0.907	1.24 (0.13, 11.66)	0.853
ɛ2/ɛ4 (ref: ɛ2/ɛ2)	**3.54 (1.12, 11.15)**	**0.031**	**3.56 (1.13, 11.25)**	**0.032**	4.12 (0.35, 48.22)	0.259
ɛ3/ɛ3 (ref: ɛ2/ɛ2)	1.50 (0.51, 4.44)	0.467	1.51 (0.51, 4.49)	0.462	1.47 (0.17, 12.95)	0.730
ɛ3/ɛ4 (ref: ɛ2/ɛ2)	**6.41 (2.16, 19.03)**	**0.001**	**6.43 (2.16, 19.15)**	**0.001**	5.03 (0.57, 44.54)	0.146
ɛ4/ɛ4 (ref: ɛ2/ɛ2)	**27.18 (8.43, 87.61)**	**<0.001**	**27.62 (8.54, 89.31)**	**<0.001**	**46.66 (3.42, 636.48)**	**0.004**
Normal weight (ref: underweight)			1.24 (0.55, 2.80)	0.605	1.22 (0.54, 2.75)	0.634
Overweight (ref: underweight)			1.20 (0.53, 2.70)	0.661	1.18 (0.53, 2.67)	0.684
Obesity (ref: underweight)			1.24 (0.55, 2.81)	0.595	1.23 (0.54, 2.78)	0.621
Married (ref: not married)			1.02 (0.88, 1.19)	0.772	1.02 (0.88, 1.20)	0.757
Education years			1.01 (0.99, 1.04)	0.337	1.01 (0.99, 1.04)	0.345
Alcohol drinking (ref: no)			1.05 (0.92, 1.21)	0.444	1.06 (0.92, 1.22)	0.427
Smoking (ref: no)			0.99 (0.54, 1.82)	0.999	1.00 (0.54, 1.83)	0.995
Psychiatric disorders (ref: no)			1.11 (0.95, 1.30)	0.214	1.11 (0.94, 1.30)	0.211
Neurological diseases (ref: no)			1.13 (0.96, 1.33)	0.147	1.13 (0.96, 1.32)	0.149
Cardiovascular diseases (ref: no)			1.05 (0.91, 1.21)	0.539	1.05 (0.90, 1.21)	0.551
Endocrinological diseases (ref: no)			1.08 (0.94, 1.24)	0.287	1.08 (0.94, 1.23)	0.305
Caffeine×female					**1.49 (1.02, 2.17)**	**0.037**
Caffeine×70–75 y					0.99 (0.65, 1.52)	0.969
Caffeine×75–80 y					1.15 (0.66, 1.98)	0.626
Caffeine×80–85 y					0.78 (0.37, 1.63)	0.510
Caffeine×ɛ2/ɛ3					0.85 (0.06, 11.30)	0.903
Caffeine×ɛ2/ɛ4					0.85 (0.05, 13.93)	0.912
Caffeine×ɛ3/ɛ3					1.05 (0.08, 12.94)	0.972
Caffeine×ɛ3/ɛ4					1.36 (0.11, 16.83)	0.813
Caffeine×ɛ4/ɛ4					0.56 (0.03, 10.54)	0.698

OR, odds ratio.

Interactions included sex×caffeine, age×caffeine, *APOE* genotypes×caffeine were further added to the multivariable logistic regression analysis to explore the effect of caffeine consumption on amyloid positivity in subgroups, and the interaction between sex×caffeine was statistically significant (OR, 1.49; 95% CI, 1.02–2.17; *p* = 0.037), while age×caffeine, and *APOE* genotypes×caffeine were not statistically significant.

### Relationship between caffeine consumption and amyloid positivity in male and female participants

The proportions of participants with amyloid positivity among caffeine and non-caffeine drinkers were 39.9% and 33.0% in male participants and 31.8% and 34.6% in female participants. Caffeine drinkers had lower ORs for elevated amyloid positivity than non-caffeine drinkers in multivariable adjusted logistic regression models (model 1: OR, 0.74; 95% CI, 0.55–0.99; *p* = 0.040; and model 2: OR, 0.72; 95% CI, 0.54–0.97; *p* = 0.032) in male participants. The association between caffeine consumption and amyloid positivity was not statistically significant among female participants. The relationship between caffeine consumption and amyloid positivity in male and female participants is presented in Table 3.

**Table 3:** Relationship between caffeine consumption and amyloid positivity in male and female participants

**Model 1**				
Factors	Male		Female	
	Adjusted OR (95% CI)	*p*	Adjusted OR (95% CI)	*p*
Caffeine consumption (ref: no)	**0.74 (0.55, 0.99)**	**0.040**	1.14 (0.90, 1.45)	0.287
70–75 y (ref: 65–70 y)	**1.48 (1.14, 1.91)**	**0.003**	**1.59 (1.31, 1.94)**	**<0.001**
75–80 y (ref: 65–70 y)	**2.58 (1.91, 3.49)**	**<0.001**	**2.08 (1.59, 2.73)**	**<0.001**
80–85 y (ref: 65–70 y)	**2.74 (1.84, 4.09)**	**<0.001**	**2.71 (1.80, 4.06)**	**<0.001**
ɛ2/ɛ3 (ref: ɛ2/ɛ2)	1.76 (0.21, 14.77)	0.602	0.83 (0.22, 3.10)	0.784
ɛ2/ɛ4 (ref: ɛ2/ɛ2)	7.09 (0.80, 62.45)	0.078	2.39 (0.61, 9.38)	0.213
ɛ3/ɛ3 (ref: ɛ2/ɛ2)	2.54 (0.31, 20.74)	0.385	1.16 (0.32, 4.17)	0.822
ɛ3/ɛ4 (ref: ɛ2/ɛ2)	**11.39 (1.39, 93.37)**	**0.023**	**4.81 (1.33, 17.35)**	**0.016**
ɛ4/ɛ4 (ref: ɛ2/ɛ2)	**50.62 (5.57, 460.14)**	**<0.001**	**19.62 (4.83, 79.61)**	**<0.001**
**Model 2**				
Factors	Male		Female	
Caffeine consumption (ref: no)	**0.72 (0.54, 0.97)**	**0.032**	1.14 (0.90, 1.46)	0.280
70–75 y (ref: 65–70 y)	**1.48 (1.14, 1.92)**	**0.003**	**1.59 (1.30, 1.95)**	**<0.001**
75–80 y (ref: 65–70 y)	**2.59 (1.91, 3.51)**	**<0.001**	**2.09 (1.58, 2.76)**	**<0.001**
80–85 y (ref: 65–70 y)	**2.73 (1.82, 4.10)**	**<0.001**	**2.74 (1.81, 4.14)**	**<0.001**
Normal weight (ref: underweight)	0.58 (0.10, 3.31)	0.537	1.43 (0.58, 3.52)	0.437
Overweight (ref: underweight)	0.53 (0.09, 3.04)	0.480	1.41 (0.57, 3.47)	0.459
Obesity (ref: underweight)	0.55 (0.09, 3.14)	0.497	1.47 (0.59, 3.65)	0.406
ɛ2/ɛ3 (ref: ɛ2/ɛ2)	1.61 (0.19, 13.68)	0.660	0.88 (0.23, 3.29)	0.847
ɛ2/ɛ4 (ref: ɛ2/ɛ2)	6.38 (0.72, 56.88)	0.097	2.56 (0.65, 10.15)	0.180
ɛ3/ɛ3 (ref: ɛ2/ɛ2)	2.33 (0.28, 19.26)	0.431	1.21 (0.33, 4.39)	0.769
ɛ3/ɛ4 (ref: ɛ2/ɛ2)	**10.45 (1.26, 86.45)**	**0.030**	**5.07 (1.40, 18.39)**	**0.014**
ɛ4/ɛ4 (ref: ɛ2/ɛ2)	**47.92 (5.22, 440.23)**	**0.001**	**21.21 (5.19, 86.65)**	**<0.001**
Married (ref: not married)	1.05 (0.78, 1.42)	0.758	1.02 (0.84, 1.22)	0.870
Education years	0.99 (0.95, 1.03)	0.716	1.02 (0.99, 1.06)	0.147
Alcohol drinking (ref: no)	1.20 (0.96, 1.50)	0.110	0.97 (0.81, 1.17)	0.783
Smoking (ref: no)	0.83 (0.37, 1.85)	0.651	1.22 (0.47, 3.13)	0.683
Psychiatric disorders (ref: no)	1.32 (0.99, 1.77)	0.060	1.02 (0.84, 1.24)	0.841
Neurological diseases (ref: no)	**1.39 (1.07, 1.80)**	**0.013**	1.00 (0.81, 1.22)	0.983
Cardiovascular diseases (ref: no)	1.06 (0.83, 1.34)	0.646	1.05 (0.87, 1.26)	0.618
Endocrinological diseases (ref: no)	1.01 (0.81, 1.26)	0.946	1.13 (0.95, 1.35)	0.177

OR, odds ratio.

### Relationship between different levels of caffeine consumption and amyloid positivity

Table 4 presents the results of different cups of caffeine consumption and amyloid positivity in all participants. No significant association was found between different cups of caffeine consumption and amyloid in all participants (model 1: OR, 1.00; 95% CI, 0.96–1.03; *p* = 0.793; and model 2: OR, 1.00; 95% CI, 0.96–1.03; *p* = 0.840). Interactions included sex×caffeine levels, age×caffeine levels, *APOE* genotypes×caffeine levels were further added to the multivariable logistic regression analysis to explore the effect of caffeine levels on amyloid positivity in subgroups, and no statistically significant interaction was found.

**Table 4:** Relationship between levels of caffeine consumption and amyloid positivity in all participants

Factors	Model 1 adjusted OR (95% CI)	*p*	Model 2 adjusted OR (95% CI)	*p*	Model 3 adjusted OR (95% CI)	*p*
Cups of caffeine consumption (ref: no)	1.00 (0.96, 1.03)	0.793	1.00 (0.96, 1.03)	0.840	0.78 (0.27, 2.28)	0.654
Female (ref: male)	1.09 (0.95, 1.25)	0.222	1.08 (0.93, 1.26)	0.292	1.05 (0.85, 1.31)	0.629
70–75 y (ref: 65–70 y)	**1.54 (1.31, 1.80)**	**<0.001**	**1.54 (1.31, 1.80)**	**<0.001**	**1.51 (1.19, 1.92)**	**0.001**
75–80 y (ref: 65–70 y)	**2.30 (1.89, 2.81)**	**<0.001**	**2.30 (1.88, 2.82)**	**<0.001**	**2.08 (1.56, 2.78)**	**<0.001**
80–85 y (ref: 65–70 y)	**2.70 (2.03, 3.58)**	**<0.001**	**2.72 (2.04, 3.63)**	**<0.001**	**2.49 (1.61, 3.83)**	**<0.001**
ɛ2/ɛ3 (ref: ɛ2/ɛ2)	1.06 (0.35, 3.21)	0.921	1.07 (0.35, 3.26)	0.905	0.83 (0.14, 4.84)	0.838
ɛ2/ɛ4 (ref: ɛ2/ɛ2)	**3.53 (1.12, 11.12)**	**0.031**	**3.54 (1.12, 11.2)**	**0.031**	2.81 (0.45, 17.59)	0.270
ɛ3/ɛ3 (ref: ɛ2/ɛ2)	1.50 (0.50, 4.44)	0.467	1.51 (0.51, 4.48)	0.462	1.16 (0.21, 6.55)	0.862
ɛ3/ɛ4 (ref: ɛ2/ɛ2)	**6.41 (2.16, 19.04)**	**0.001**	**6.42 (2.15, 19.12)**	**0.001**	4.56 (0.81, 25.68)	0.085
ɛ4/ɛ4 (ref: ɛ2/ɛ2)	**27.16 (8.42, 87.56)**	**<0.001**	**27.54 (8.52, 89.08)**	**<0.001**	**28.21 (4.33, 183.79)**	**<0.001**
Normal weight (ref: underweight)			1.24 (0.55, 2.79)	0.609	1.24 (0.55, 2.80)	0.600
Overweight (ref: underweight)			1.19 (0.53, 2.69)	0.669	1.20 (0.53, 2.71)	0.658
Obesity (ref: underweight)			1.24 (0.55, 2.80)	0.611	1.24 (0.55, 2.82)	0.602
Married (ref: not married)			1.02 (0.88, 1.19)	0.781	1.03 (0.88, 1.20)	0.739
Education years			1.01 (0.99, 1.04)	0.337	1.01 (0.99, 1.04)	0.346
Alcohol drinking (ref: no)			1.05 (0.91, 1.20)	0.521	1.05 (0.91, 1.20)	0.530
Smoking (ref: no)			0.99 (0.54, 1.82)	0.973	0.98 (0.53, 1.81)	0.955
Psychiatric disorders (ref: no)			1.11 (0.94, 1.30)	0.208	1.11 (0.94, 1.30)	0.209
Neurological diseases (ref: no)			1.13 (0.96, 1.33)	0.131	1.13 (0.96, 1.32)	0.141
Cardiovascular diseases (ref: no)			1.05 (0.91, 1.21)	0.521	1.04 (0.90, 1.21)	0.555
Endocrinological diseases (ref: no)			1.08 (0.94, 1.24)	0.291	1.08 (0.94, 1.24)	0.303
Cups of caffeine×female					1.01 (0.94, 1.09)	0.712
Cups of caffeine×70–75 y					1.01 (0.93, 1.10)	0.822
Cups of caffeine×75–80 y					1.05 (0.95, 1.16)	0.318
Cups of caffeine×80–85 y					1.05 (0.88, 1.24)	0.591
Cups of caffeine×ɛ2/ɛ3					1.23 (0.42, 3.59)	0.708
Cups of caffeine×ɛ2/ɛ4					1.22 (0.41, 3.63)	0.725
Cups of caffeine×ɛ3/ɛ3					1.23 (0.42, 3.58)	0.702
Cups of caffeine×ɛ3/ɛ4					1.28 (0.44, 3.72)	0.650
Cups of caffeine×ɛ4/ɛ4					1.09 (0.37, 3.26)	0.872

OR, odds ratio.

## DISCUSSION

The present study found no significant association between caffeine consumption and amyloid positivity in all participants. Subgroup analysis showed that caffeine consumption may be inversely associated with amyloid positivity, predominantly among males but not females. Moreover, caffeine consumption level was not associated with amyloid positivity. These findings suggest that caffeine consumption is associated with a reduced pathological cerebral amyloid positivity in older male adults, possibly through its contribution to lowering the risk of AD or related cognitive decline.

The null association between caffeine consumption and amyloid positivity in all participants in this study was not consistent with previous findings, which indicated that high caffeine consumption is associated with lower amyloid accumulation [23]. Similar to studies that explored the association between caffeine consumption and other health outcomes (cognitive decline or dementia), the effect of caffeine did not seem to be robust. As indicated in the literature [10], the effect of caffeine was generally affected by the chronicity of exposure, categories, and doses of caffeine-containing beverages, as well as the characteristics of drinkers, which possibly explains the discrepancy in findings between studies.

In the meantime, an explicit explanation for the effect of caffeine consumption on amyloid burden in older male adults needs to be explored in future research. Preclinical studies on male aged rats have supported that caffeine intake contributes to reducing pathological cerebral amyloid deposition, possibly by mitigating Aβ-induced antioxidant activity [33, 34], inhibiting enzymes for Aβ production [17, 35, 36], and reducing Aβ-induced mitochondrial dysfunction [37, 38]. However, the precise neuronal cellular mechanisms underlying sex differences in caffeine and amyloid burdens should be further explored in the future.

The present findings also provide a neuropathological explanation for the negative association between caffeine and lower risk of AD or cognitive decline in males [11, 13]. Several meta-analyses have supported caffeine consumption as a protective factor against dementia or cognitive impairment [39–41], while a few studies have shown that caffeine may not be effective in delaying cognitive decline [42]. Whether there are sex-specific effects of caffeine consumption on cognitive impairment remains unclear. The inconclusive results regarding caffeine and cognitive impairment may be explained by the characteristics of different individuals since we found that caffeine intake had a distinct effect on amyloid positivity in participants with different demographic characteristics (e.g., sex, age, and BMI). Although the current findings provided an explanation for the association between caffeine intake and reduced cognitive decline in men, the results that support the benefits of caffeine in women have not been well interpreted [43]. Further results from well-designed and well-conducted cohort studies are required to derive robust evidence regarding caffeine consumption and cognitive indicators in male and female participants with different demographic characteristics.

Dose-specific analysis also suggested that no significant association between different levels of caffeine consumption and amyloid positivity. The association between doses of caffeine intake and health outcomes remained debatable. Pervious findings indicated that moderate intake was the most efficacious [13, 44]; nevertheless, two studies have pointed that high level of caffeine consumption could reduce amyloid pathology [18, 19]. As for the amount of caffeine consumed daily, noteworthily, excessive caffeine consumption can cause caffeine poisoning, the symptoms of which include anxiety, agitation, insomnia, gastrointestinal disorders, and mental disorders [45]. Thus, the effects of different amount caffeine on amyloid pathology need further investigated.

The strength of this study is its large population-based sample size; however, several limitations exist. First, the self-reported number of cups of caffeine-containing beverages per day and lack of clarity of chronicity of exposure to caffeine consumption may not reflect the actual magnitude of caffeine intake. Thus, the association between different levels of caffeine consumption and amyloid positivity should be cautiously interpreted as measurement errors may occur. Second, this study was limited to participants aged >65 years without cognitive impairment, thereby limiting the analyses to the outcome of the emerging Aβ pathology in the absence of significant cognitive dysfunction. Third, the vast majority of A4 study participants were white and not representative of the population at risk for AD. Fourth, this is a cross-sectional study. Therefore, associations between caffeine consumption and amyloid positivity cannot necessarily be considered causal relationships. Fifth, the current results should be cautiously read, as it merely reflects the association between caffeine consumption and amyloid positivity, while no significant sex-specific effect on the association of caffeine consumption and amyloid levels using continuous measures.

### Conclusions

The present study found no significant association between caffeine consumption and amyloid positivity in the whole participants. Caffeine consumption was inversely associated with amyloid positivity predominantly among males, but not females. Well-established studies are required to explore and validate the effects of caffeine consumption on brain amyloid positivity in the future.

## Supplementary Material

Supplementary Material

## Data Availability

The data used in this study were extracted from the Anti-Amyloid Treatment in Asymptomatic Alzheimer’s (A4)/Longitudinal Evaluation of Amyloid Risk and Neurodegeneration (LEARN) Study.
