# Supplementary material for: Association of Caffeine Consumption and Brain Amyloid Positivity in Cognitively Normal Older Adults
Source: J Alzheimers Dis. 2023 May 16;93(2):483–93. doi: 10.3233/JAD-220591 (PMC10200149; doi:10.3233/JAD-220591)
Supplement: Supplementary Material [file jad-93-jad220591-s001.pdf]

Supplementary Material

Association of Caffeine Consumption and Brain Amyloid Positivity in Cognitively Normal Older Adults

Supplementary Table 1. Summary of the questions related to this study.

**Questions**	**Assessment/ measures**	**Types of covariates**	**Choice**
Average number of cups of caffeine consumed per day:	Questionnaire	Continuous variables	
		Categorical variables	Yes, No
Sex:	Questionnaire	Categorical variables	Male, Female
Age at screening visit:	Questionnaire	Continuous variables	
		Categorical variables	65–70, 70–75, 75–80, 80–85
Body mass index:	Physically measured	Continuous variables	
		Categorical variables	<18.5, 18.5–24.9, 25.0–29.9, ≥30.0
Marital status	Questionnaire	Categorical variables	Married, Not married (widowed; divorced; never married; unknown)
Education (years):	Questionnaire	Continuous variables	
Racial categories:	Questionnaire	Categorical variables	American Indian or Alaskan Native, Asian, Native Hawaiian or Other Pacific Islander, Black or African American, White, Unknown or not reported
Average number of alcoholic drinks consumed per day:	Questionnaire	Continuous variables	
		Categorical variables	Yes, No
Average number of packs smoked per day:	Questionnaire	Continuous variables	
		Categorical variables	Yes, No
APOE4 Genotype:	Genotype	Categorical variables	ε2/ε2, ε2/ε3, ε2/ε4, ε3/ε3, ε3/ε4, ε4/ε4, Not available
Is symptom/condition ongoing? (Psychiatric)	Questionnaire	Categorical variables	Yes, No
Is symptom/condition ongoing? (Neurologic (other than AD))	Questionnaire	Categorical variables	Yes, No
Is symptom/condition ongoing? (Cardiovascular)	Questionnaire	Categorical variables	Yes, No
Is symptom/condition ongoing? (Endocrine-Metabolic)	Questionnaire	Categorical variables	Yes, No

APOE, apolipoprotein E.
